# YAP/TAZ inhibition reduces metastatic potential of Ewing sarcoma cells

**DOI:** 10.1038/s41389-020-00294-8

**Published:** 2021-01-08

**Authors:** Lisa Bierbaumer, Anna M. Katschnig, Branka Radic-Sarikas, Maximilian O. Kauer, Jeffrey A. Petro, Sandra Högler, Elisabeth Gurnhofer, Gloria Pedot, Beat W. Schäfer, Raphaela Schwentner, Karin Mühlbacher, Florian Kromp, Dave N. T. Aryee, Lukas Kenner, Aykut Uren, Heinrich Kovar

**Affiliations:** 1 Children’s Cancer Research Institute, Zimmermannplatz 10, 1090 Vienna, Austria; 2 Georgetown University Medical Center, Lombardi Comprehensive Cancer Center, Washington, DC USA; 3 Institute of Pathology, Unit of Laboratory Animal Pathology, University of Veterinary Medicine Vienna, Veterinärplatz 1, Vienna, Austria; 4 Department of Pathology, Medical University of Vienna, Vienna, Austria; 5 Department of Oncology and Children’s Research Center, University Children’s Hospital, Zurich, Switzerland; 6 Department of Paediatrics, Medical University Vienna, Vienna, Austria; 7 Christian Doppler Laboratory for Applied Metabolomics (CDL-AM), Währingergürtel 18-20, Medical University Vienna, Vienna, Austria; 8 CBmed Vienna, Corelab 2, Währingergürtel 18-20, Medical University Vienna, Vienna, Austria

**Keywords:** Paediatric cancer, Focal adhesion

## Abstract

Ewing sarcoma (EwS) is a highly metastatic bone cancer characterized by the ETS fusion oncoprotein EWS-FLI1. EwS cells are phenotypically highly plastic and switch between functionally distinct cell states dependent on EWS-FLI1 fluctuations. Whereas EWS-FLI1^high^ cells proliferate, EWS-FLI1^low^ cells are migratory and invasive. Recently, we reported activation of MRTFB and TEAD, effectors of RhoA and Hippo signalling, upon low EWS-FLI1, orchestrating key steps of the EwS migratory gene expression program. TEAD and its co-activators YAP and TAZ are commonly overexpressed in cancer, providing attractive therapeutic targets. We find TAZ levels to increase in the migratory EWS-FLI1^low^ state and to associate with adverse prognosis in EwS patients. We tested the effects of the potent YAP/TAZ/TEAD complex inhibitor verteporfin on EwS cell migration in vitro and on metastasis in vivo. Verteporfin suppressed expression of EWS-FLI1 regulated cytoskeletal genes involved in actin signalling to the extracellular matrix, effectively blocked F-actin and focal-adhesion assembly and inhibited EwS cell migration at submicromolar concentrations. In a mouse EwS xenograft model, verteporfin treatment reduced relapses at the surgical site and delayed lung metastasis. These data suggest that YAP/TAZ pathway inhibition may prevent EwS cell dissemination and metastasis, justifying further preclinical development of YAP/TAZ inhibitors for EwS treatment.

## Introduction

Ewing sarcoma (EwS), the second most common malignant bone tumour in children and adolescents, has a high propensity for early onset dissemination, and current treatment strategies are only poorly effective against metastatic disease^1^. EwS is driven by a *EWSR1-ETS* fusion oncogene, most commonly *EWSR1-FLI1*^2,3^, which results in the expression of the oncogenic transcription factor EWS-FLI1 that drives cell transformation and oncogenicity^4^. Recent studies suggest that EWS-FLI1 may oscillate between high and low expression states, thereby orchestrating distinct phenotypic programs^5^. The majority of tumour cells express high EWS-FLI1 levels, proliferate and exhibit a high cell-cell adhesion propensity. In contrast, rare EWS-FLI1^low^ cells resemble the putative EwS cell of origin, mesenchymal stem cells (MSCs)^6^, are highly migratory^5,7,8^ and more efficient in forming metastases as compared to EWS-FLI1^high^ cells^5,9^.

We have recently identified a regulatory mechanism involving the myocardin related and TEA-domain transcription factors MRTFB and TEAD1–4 triggering cytoskeletal reorganization in EWS-FLI1^low^ cells^10^. In contrast, in EWS-FLI1^high^ cells, EWS-FLI1 bound to and prohibited access of MRTFB to TEAD-regulated enhancers. TEADs require co-activation by the Yes-associated protein 1 (YAP-1, YAP) or its paralogue, the transcriptional co-activator with PDZ-binding motif (TAZ, WWTR1), which are typically controlled by Hippo signalling in organ development and tissue homoeostasis^11,12^. Independent of the canonical Hippo pathway, mechano-skeletal dynamics involving the Rho signalling pathway has been shown to control MRTFB and YAP/TAZ activity^11,13,14^. Overexpression and hyperactivation of YAP and/or TAZ have been linked to cancer growth and metastasis in various tumours^15–20^. However, somatic or germline mutations of Hippo signalling components are uncommon in human cancers including EwS^21,22^, suggesting other regulatory mechanisms of YAP/TAZ activation. Mechanistically, YAP has been reported to be constitutively stabilized by the polycomb group family protein BMI-1 and to be required for EwS cell proliferation and loss of contact inhibition^23^. The role of TAZ in the context of EwS remained elusive.

As YAP/TAZ activity is low in resting tissues, pharmacological inhibition of the YAP/TAZ/TEAD axis might provide a novel therapeutic strategy to prevent metastasis without severe side effects^24^. Depletion of YAP and TAZ reversed epithelial-to-mesenchymal transition (EMT) and blocked migration and metastatic propensity of a variety of cell types^25,26^. Previously, the photoactive porphyrin verteporfin (VP), a clinically approved drug (Visudyne) for treatment of age-related macular degeneration, showed high specificity in preventing complex formation of YAP with TEAD and subsequent target gene activation, independent of its photosensitizing effect^20,27^. Since VP treatment prohibited cell migration in various cancers^28,29^, we aimed at testing its effects on early steps of EwS metastasis.

## Results

### TAZ expression is repressed by EWS-FLI1 in vitro and negatively correlates with EWS-FLI1 in tumours

**Fig. 1:**
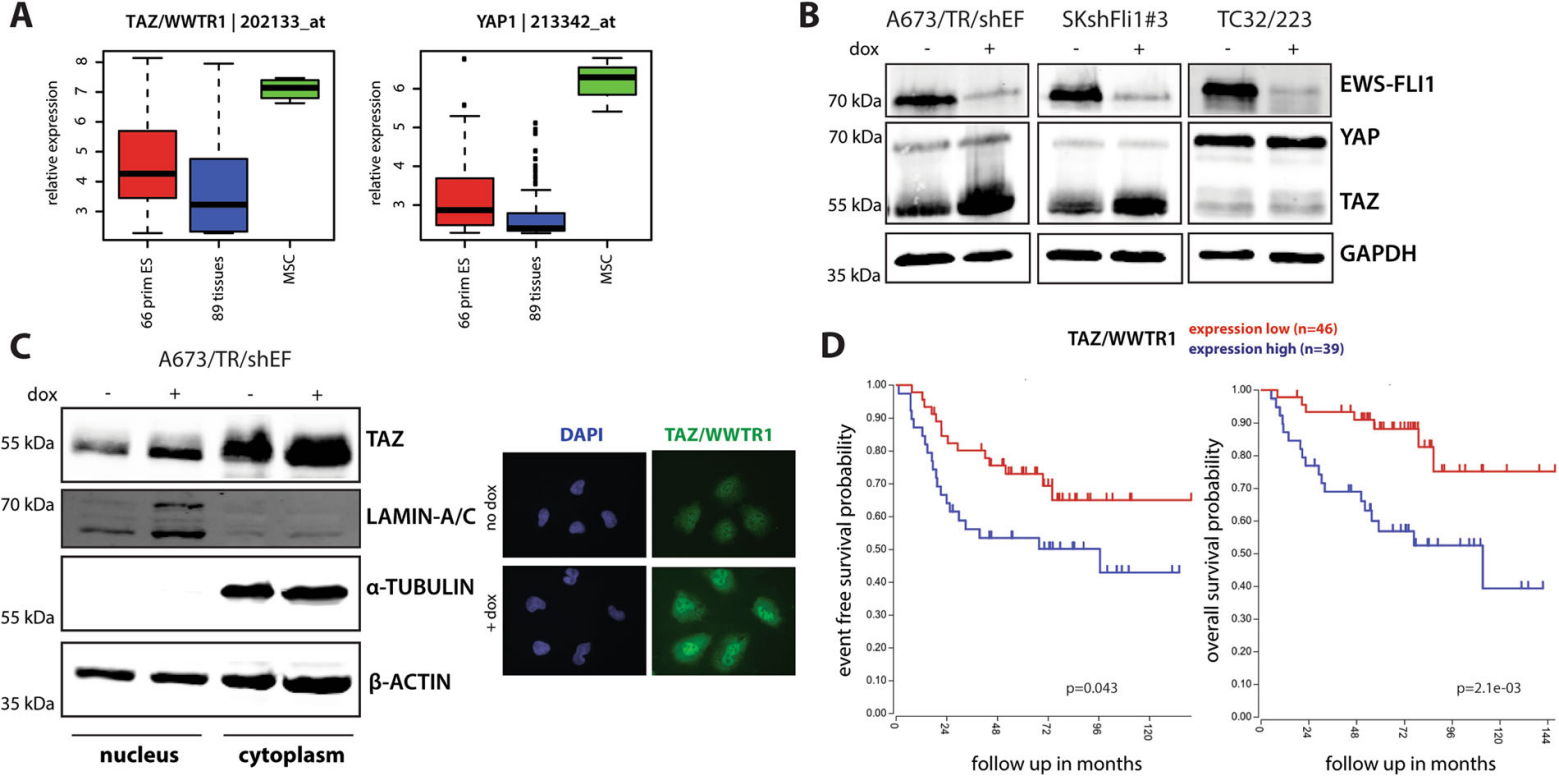
TAZ expression is repressed by EWS-FLI1 in vitro and correlates with patient outcome in primary EwS. **a** Box-plots showing relative expression levels of TAZ and YAP in 66 primary EwS tumours compared to 89 normal tissues and mesenchymal stem cells (MSC). Affymetrix hgu133a microarray expression data from Kauer et al.^61^. **b** Representative immunoblots showing YAP and TAZ expression under dox-induced EWS-FLI1^low^ (48 h dox) versus EWS-FLI1^high^ conditions in different EwS cell lines. **c** Subcellular localization of TAZ in A673/TR/shEF cells upon EWS-FLI1^high^ (no dox) and EWS-FLI^low^ (48 h dox) conditions was analysed by protein fractionation experiments and immunofluorescence microscopy. One representative immunoblot out of three biological replicates is shown. Lamin A/C and α-tubulin were used as nuclear and cytoplasmic fraction controls, respectively. Immunofluorescent pictures are shown at ×40 magnification. **d** Kaplan–Meier analysis of huex10t microarray expression data for event-free (chi = 4.08, *p* = 0.043; left graph) and overall (chi = 9.44, *p* = 2.1e−03; right graph) survival of 85 EwS patients in relation to TAZ expression. Data (GEO ID: gse63157^33^) was visualized using the R2 database (http://r2.amc.nl).

To test for the expression of YAP and TAZ in EwS, we compared 66 primary EwS with 89 normal tissues. The expression of both genes was moderately increased in the EwS context (Fig. 1a). In line with their important role as regulators of MSC fate^30,31^, YAP and TAZ expression was specifically high in MSCs, the presumed EwS cell type of origin (Fig. 1a).

As EWS-FLI1^low^ expressing cells have been linked to an invasive phenotype recently, we asked whether EWS-FLI1 affects YAP and TAZ protein levels in three EwS cell lines carrying doxycycline (dox) - inducible sh-EWS-FLI1 constructs. In general, YAP and TAZ expression levels varied between cell lines (Fig. 1b). Upon EWS-FLI1 knockdown, TAZ substantially increased in A673/TR/shEF and SKshFli1#3 cells but not in TC32/223 cells. By immunofluorescence microscopy and protein fractionation of A673/TR/shEF cells, TAZ was found to accumulate in the cytoplasm and the nucleus, and to increase in both compartments upon EWS-FLI1 modulation (Fig. 1c). In line with EWS-FLI1 negatively regulating TAZ, an analysis of RNA expression data of 117 primary EwS tumours (GEO ID: gse34620^32^) revealed significant anti-correlation of TAZ expression with FLI1 (*r* = −0.225, *p* = 0.015) and with selected EWS-FLI1 activated genes (BCL11B: *r* = −0.539, *p* = 3.49e−10; NKX2–2: *r* = −0.448, *p* = 3.95e−07), as well as a positive correlation with EWS-FLI1 suppressed genes (TGFBR2: *r* = 0.819, *p* = 1.75e−29; LOX: *r* = 0.752, *p* = 0.166e−22) (Supplemental Fig. 1). In contrast, YAP did not significantly correlate with FLI1 or EWS-FLI1 targets (data not shown). Furthermore, Kaplan–Meier analysis of an independent series of 85 primary EwS with complete clinical annotation (GEO ID: gse63157^33^) indicated an association of high TAZ expression with adverse event-free (chi = 4.08, *p* = 0.043) and overall survival (chi = 9.44, *p* = 2.1e−03) (Fig. 1d). These findings suggest that TAZ might play an important role in the onset of EwS metastasis.

### Verteporfin prevents complex formation of YAP/TAZ with TEAD upon low EWS-FLI1

**Fig. 2:**
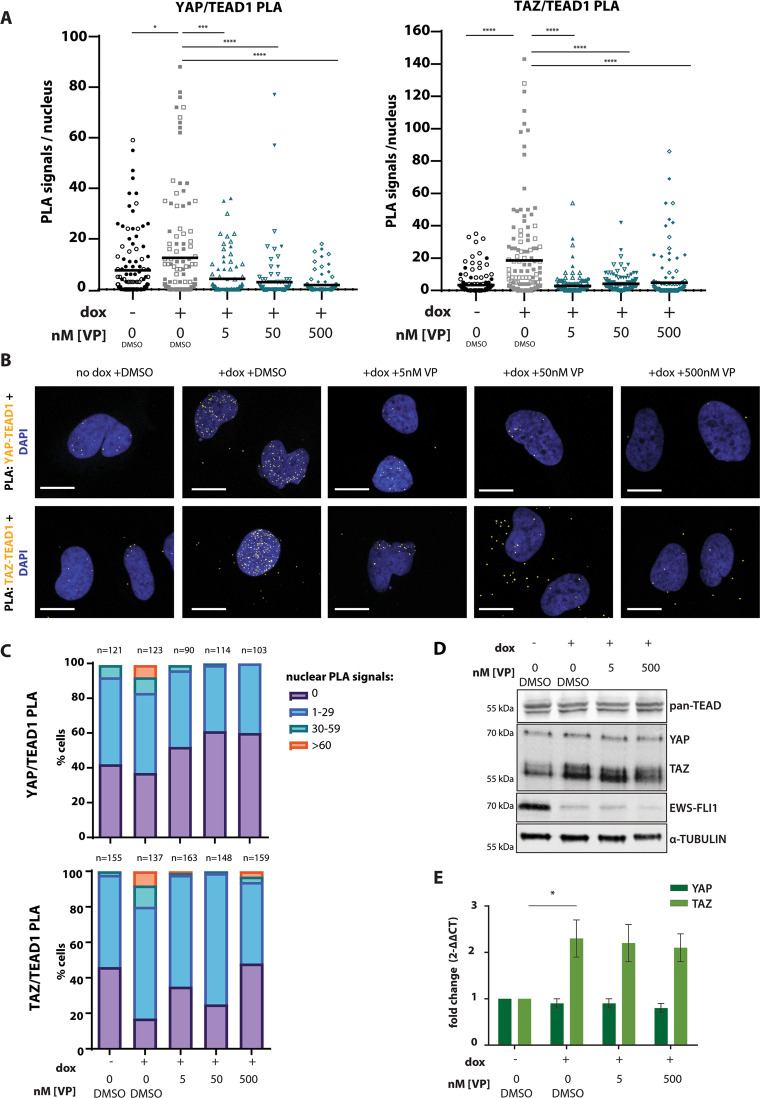
Verteporfin prevents complex formation of YAP/TAZ and TEAD in A673/TR/shEF. **a** Quantification of nuclear YAP/TEAD1 and TAZ/TEAD1 PLA signals under EWS-FLI1^high^ (no dox) and EWS-FLI1^low^ ( + dox) conditions and upon VP (5 nM, 50 nM, 500 nM) treatment. Pooled data from two independent biological replicates, represented by distinct symbol patterns, is shown. Mean numbers of PLA signals/cell are indicated. (**b**) Representative confocal images (63x objective, zoom factor 2.5) of nuclear YAP/TEAD1 and TAZ/TEAD1 PLA signals from experiments shown in (A). Scale bar: 20 µm. (**c**) Quantification of YAP/TEAD1 and TAZ/TEAD1 PLA signals shown in (A), represented as % of cells with corresponding PLA signal ranges per nucleus. (**d**) Immunoblot showing expression of pan-TEAD, YAP/TAZ and EWS-FLI1 from total protein lysates upon EWS-FLI1^high^ (no dox) and EWS-FLI1^low^ ( + dox) conditions and upon VP treatment. One representative experiment from three biological replicates is shown. (**e**) qPCR analysis of YAP and TAZ mRNA transcripts upon same experimental conditions as in (D). Expression values are shown as fold change ± s.e.m of three biological replicates relative to no dox +DMSO-control conditions. All statistics were calculated by two-sided, unpaired Student’s t-test *p ≤ 0.05, **** p ≤ 0.0001.

We previously demonstrated constitutive nuclear localization of YAP and increased association with TEAD-regulated enhancers upon EWS-FLI1 knockdown^10^. To test if EWS-FLI1 depletion also directly affected the complex formation of TEAD with its co-activators YAP and TAZ, we performed proximity ligation assays (PLAs), which allow antibody-based in situ visualization of protein–protein interactions at single-molecule and single-cell resolution. Quantification of nuclear PLA signals revealed a significant increase of endogenous YAP/TEAD1 as well as TAZ/TEAD1 interactions in the EWS-FLI1^low^ compared to the EWS-FLI1^high^ state (Fig. 2a, b). Apparently, only minor subpopulations of cells showed very high numbers of PLA signals (i.e. >60 PLA dots per nucleus), suggesting heterogeneity of YAP/TAZ/TEAD transcriptional activity at single-cell level. However, the proportion of cells with no YAP/TEAD1 and TAZ/TEAD1 PLA signals was lower while the proportion of cells with >30 PLA signals was higher in the EWS-FLI1^low^ than the EWS-FLI1^high^ state (Fig. 2c). Correspondingly, increased pulldown of YAP and TAZ was observed in pan-TEAD co-immunoprecipitation (co-IP) experiments upon EWS-FLI1 knockdown and, vice versa, TEAD was enriched in TAZ precipitates in the EWS-FLI1^low^ state (Supplemental Fig. 2A). Functional activation of YAP/TAZ/TEAD upon EWS-FLI1^low^ conditions was confirmed by increased expression of YAP/TAZ/TEAD target genes *CYR61, CTGF* and *SERPINE1* (Supplemental Fig. 2B). Our data highlight enhanced activation of the YAP/TAZ/TEAD signalling axis as a specific property of EWS-FLI1^low^ cells.

Next, we evaluated the consequences of treatment with verteporfin (VP), an established pharmacological YAP/TAZ inhibitor, on EwS biology. VP was reported to perturb YAP/TAZ function; however, the exact mode of its activity is controversial. To test whether VP affects YAP/TAZ/TEAD complex formation, we conducted PLAs in A673/TR/shEF EWS-FLI1^low^ cells treated with different VP concentrations. Importantly, all VP experiments were protected from ambient light to avoid generation of reactive oxygen species (ROS) and unspecific cell toxicity. VP treatment at concentrations as low as 5 nM were sufficient to significantly reduce the mean number of nuclear YAP/TEAD1 and TAZ/TEAD1 PLA signals, to increase the percentage with no detectable YAP/TAZ/TEAD1 interactions and to lower the number of cells with high PLA signal numbers (Fig. 2a-c). In line with our PLA results, VP treatment inhibited the expression of YAP/TAZ target genes (Supplemental Fig. 2B) and strongly decreased YAP and TAZ co-precipitation in the pan-TEAD co-IP assays (Supplemental Fig. 2A). Conversely, TEAD was reduced in YAP and TAZ pulldown assays (Supplemental Fig. 2A). Neither expression levels of TEADs nor of YAP or TAZ were significantly affected on protein (Fig. 2d) or RNA level (Fig. 2e) by VP treatment. Similarly, YAP and TAZ levels remained unaffected by VP under EWS-FLI1^high^ conditions (Supplemental Fig. 3A, B). In summary, our data highlight VP’s function as a potent YAP/TAZ/TEAD complex suppressor in EwS.

### Verteporfin prohibits EwS cell migration and invasion in vitro

**Fig. 3:**
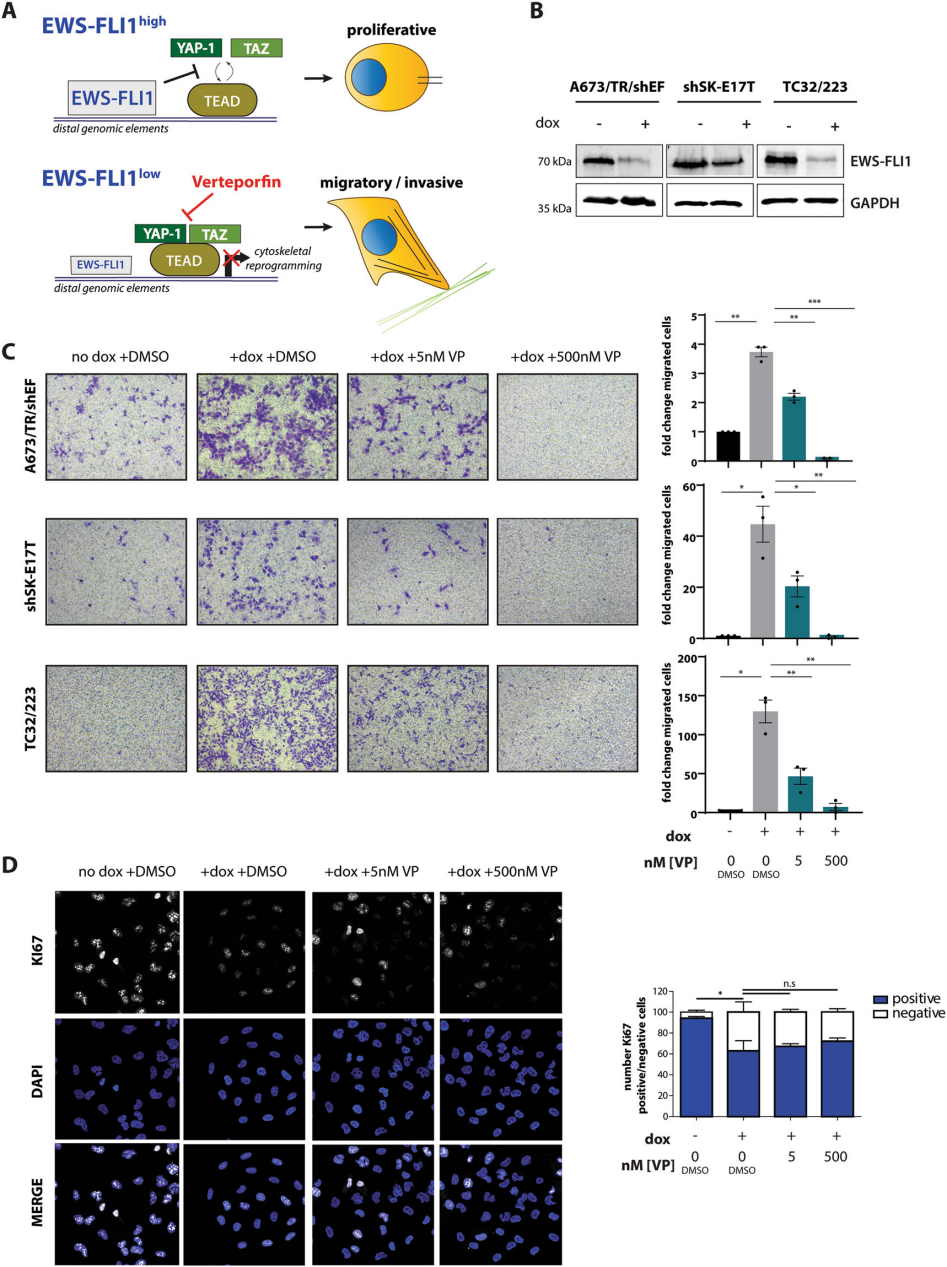
Verteporfin inhibits cell migration of EWS-FLI1^low^ cells without affecting proliferation. **(a)** Proposed model of YAP/TAZ/TEAD pathway inhibition by VP in EWS-FLI1^low^ cells and its potential effect on EwS cell phenotype. **(b)** Representative immunoblots verifying dox-induced knockdown of EWS-FLI1 in A673/TR/shEF, shSK-E17T and TC32/223 cell lines at the start of the migration assay. **(c)** Boyden chamber migration assays using cell lines from (B) in presence and absence of different VP concentrations. Exemplary pictures of migrated cells as well as quantification (fold change of migrated cells relative to “no dox +DMSO”) of three biological replicates performed in triplicates are shown. **(d)** Cell proliferation of A673/TR/shEF cells was evaluated by KI67 positivity in parallel to Boyden chamber assays. KI67 positive and negative fractions from three biological replicates were determined and normalized to the total number of counted cells. Statistics were calculated by One-sample t-test for comparison to “no dox +DMSO” conditions and two-sided, unpaired Student’s t-test for all other conditions. *p ≤ 0.05, **p ≤ 0.01, ***p ≤ 0.001.

As YAP and TAZ association with TEAD was increased under EWS-FLI1^low^ conditions, we hypothesised that pharmacologic inhibition of YAP/TAZ/TEAD complex formation by VP may prevent EwS cells from pro-invasive cytoskeletal reprogramming in EWS-FLI1^low^ cells and thus potentially suppress EwS metastasis (Fig. 3a). Therefore, we performed Boyden chamber migration assays upon EWS-FLI1^high^ and EWS-FLI1^low^ conditions in absence and presence of VP (Fig. 3b, c). EwS cells migrated strongly, especially when expression of the fusion oncogene was low. In contrast, VP treatment at concentrations as low as 5 nM significantly reduced the migratory capacity of EWS-FLI1^low^ cells. Treatment with 500 nM VP completely abolished EwS cell migration in A673/TR/shEF and shSK-E17T cells and strongly inhibited migration of TC32/223 cells. Similar results were obtained in another EwS cell line, TC71, transiently transfected with sh-EWS-FLI1, further corroborating the anti-migratory activity of VP treatment (Supplemental Fig. 4A). VP is light sensitive, and 10 minutes exposure of EwS cells treated with 500 nM VP to ambient laboratory artificial light conditions during handling sufficed to induce strong ROS production (Supplemental Fig. 4B). Although all our VP experiments were performed under conditions protected from artificial light illumination with almost no ROS production (Supplemental Fig. 4B), we repeated migration assays with A673/TR/shEF cells in complete darkness to exclude any ROS-dependent effects. In complete darkness, low VP concentrations exerted comparable anti-migratory activity as described for our standard light-protected experimental conditions, while the anti-migratory effect of 500 nM VP on EWS-FLI1^low^ cells was slightly reduced to the level of EWS-FLI1^high^ cells (Supplemental Fig. 4C). These results suggest that low-level ROS may contribute to a YAP/TAZ-independent anti-migratory effect at high VP concentrations while the anti-migratory activity of low nanomolar VP concentrations was independent of ROS and likely due to interference with YAP/TAZ/TEAD signalling.

Further, we tested for VP’s anti-invasive properties in tumour spheroid collagen invasion assays. VP strongly prohibited invasion of EWS-FLI1^low^ cells into the surrounding matrix in a concentration-dependent manner (Supplemental Fig. 4D). To rule out a potential effect of VP on proliferation, we monitored the fraction of KI67 positive cells by immunofluorescence microscopy. Knockdown of EWS-FLI1 significantly reduced KI67 positive counts and treatment with VP did not further decrease this number (Fig. 3d). In addition, anti-migratory VP concentrations were significantly lower than the corresponding IC_50_ values for each of the tested EwS cell lines at the time range of the motility assays (24–72 h; Supplemental Fig. 5).

**Fig. 4:**
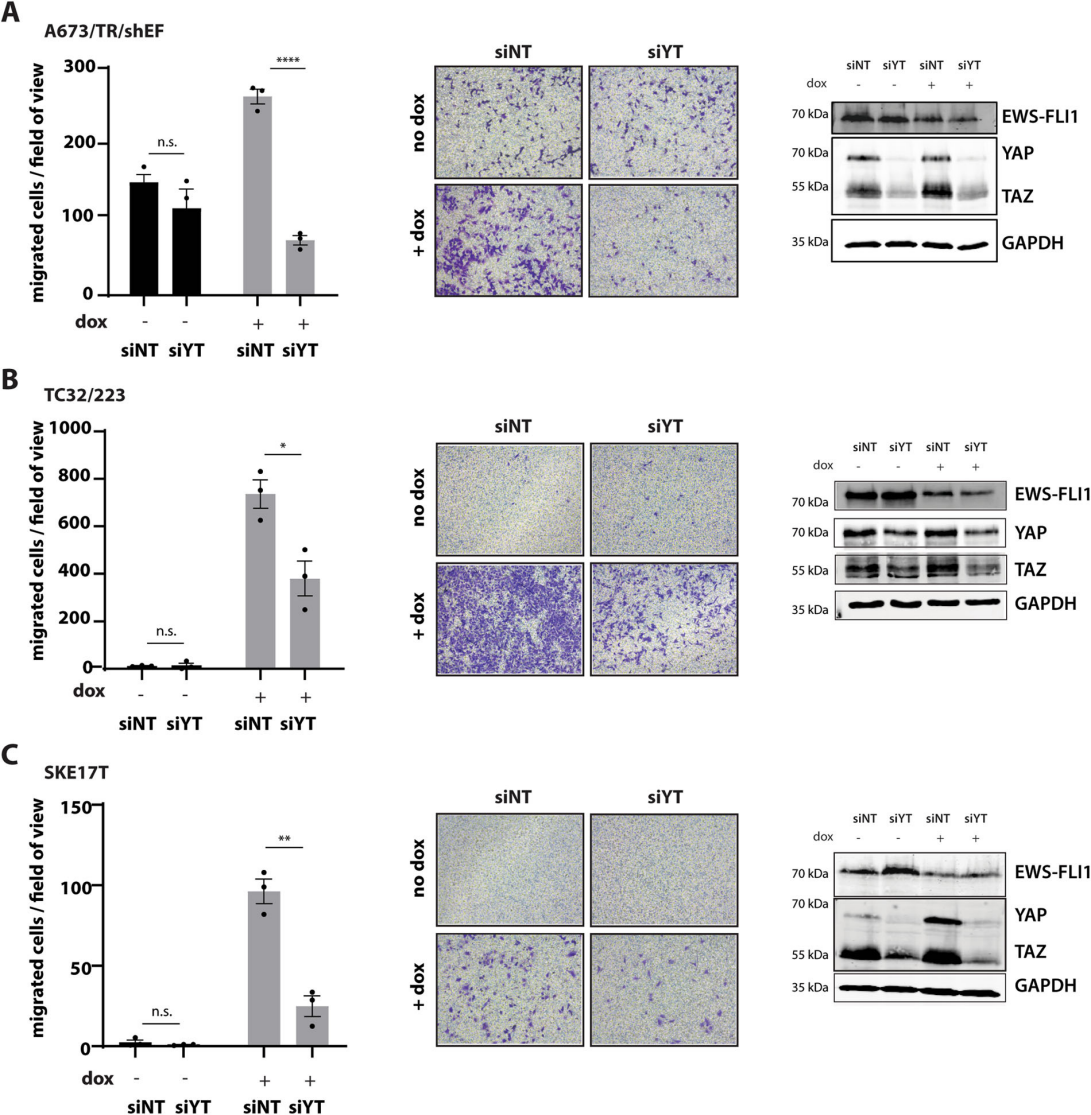
Combined silencing of YAP/TAZ interferes with the migratory capacity of EWS-FLI1^low^ cells, similar to Verteprofin treatment. Boyden chamber migration assays upon combined knockdown of YAP/TAZ (siYT), compared to cells transfected with control siRNA (siNT), under EWS-FLI1^high^ and dox-induced EWS-FLI1^low^ conditions in **(a)** A673/TR/shEF, **(b)** TC32/223 and **(c)** shSK-E17T. Efficient silencing of YAP/TAZ and EWS-FLI1 knockdown are shown by one representative immunoblot. Data are represented as mean ± s.e.m. of three biological replicates performed in triplicates. Statistics were calculated by two-sided, unpaired Student’s t-test *p ≤ 0.05, **p ≤ 0.01, ***p ≤ 0.001.

We next aimed for reproducing the anti-migratory effect of VP with genetic silencing of YAP and TAZ. Therefore, we interrogated the phenotypic consequences of combined YAP/TAZ knockdown under EWS-FLI1^low^ and EWS-FLI1^high^ conditions. Similar to VP treatment, siRNA-mediated depletion of YAP and TAZ resulted in reduced EwS cell migration in three EwS cell lines, especially under EWS-FLI1^low^ conditions (Fig. 4a-c). While individual silencing of YAP did not suffice to inhibit migration of EWS-FLI1^low^ cells significantly, depletion of TAZ reduced the number of migrated cells to levels comparable with those at EWS-FLI1^high^ conditions (Supplemental Fig. 6). This may suggest different roles for YAP and TAZ in mediating the migratory potential of EwS cells.

### Verteporfin affects cytoskeletal gene expression of EwS cells

**Fig. 5:**
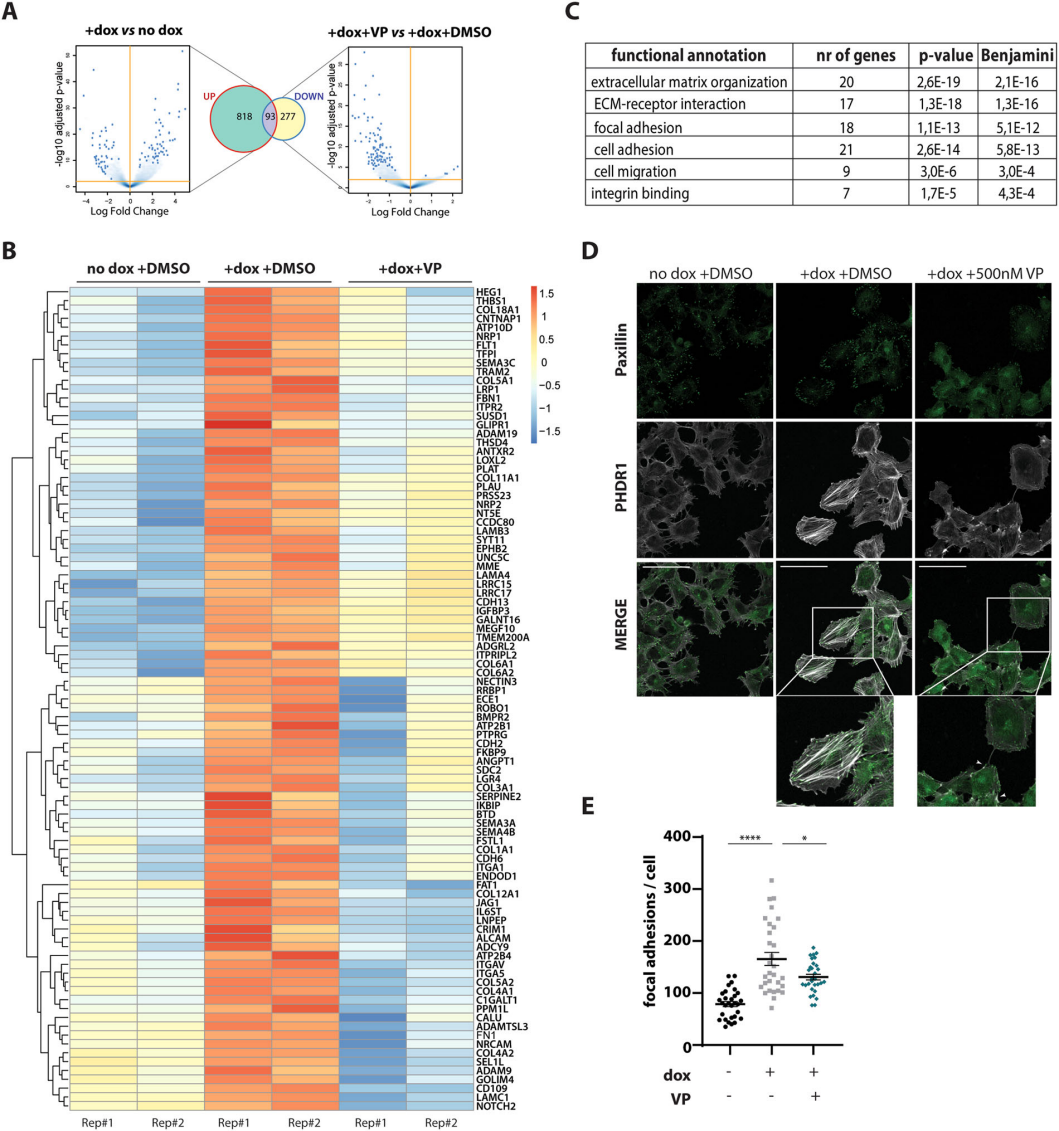
Verteporfin affects expression of cytoskeletal genes leading to reduced F-actin and focal-adhesion formation in A673/TR/shEF. **(a)** Volcano plots and Venn diagrams showing gene expression upon EWS-FLI1^low^ versus EWS-FLI1^high^ ( + dox *vs* no dox) conditions compared to the effects of 500 nM VP treatment versus DMSO control (+dox+VP *vs* + dox+DMSO) in the EWS-FLI1^low^ state. 911 genes were found to increase (Venn diagram: UP) in expression upon EWS-FLI1^low^ versus EWS-FLI1^high^ conditions (EWS-FLI1-anticorrelated genes). Treatment with VP downregulated gene expression (Venn diagram: DOWN) of a total of 370 genes, of these 93 genes are EWS-FLI1-anticorrelated genes. Cells were collected in parallel to performing transwell migration assays (see Fig. 3), and RNA from two biological replicates was sent for RNA-seq. Volcano plots show log2-fold changes of gene expression, Venn diagrams show genes changing with a │logFC│>0.7, p < 0.05. **(b)** Heatmap showing effects of 500 nM VP on EWS-FLI1-anticorrelated genes of two biological replicates (Rep#1,2). **(c)** DAVID functional annotation analysis of genes shown in Fig. 5b. EWS-FLI1-anticorrelated genes, which were affected by VP treatment (93 genes) are significantly enriched in cytoskeletal processes, such as communication with the extracellular matrix, adhesion and migration. **(d)** Confocal immunofluorescence microscopy showing effects of 500 nM VP treatment on stress fibre (TRITC-phalloidin, PHDR1) and focal-adhesion (FITC-paxillin) formation. F-actin thickness and abundance is increased in the migratory EWS-FLI1^low^ ( + dox) state as compared to EWS-FLI1^high^ (no dox). Number and intensity of focal adhesions is also strongly increased. Treatment with VP reduced the number of focal adhesions at the leading cell edges and led to F-actin breakdown (indicated with arrows). Representative confocal images from two biological replicates are shown (40x magnification, selection: zoom 146%, scale bar: 100 µm). **(e)** Quantification of paxillin-positive foci, normalized to respective cell size, in the migratory EWS-FLI1^low^ ( + dox; ±500 nM VP) state and compared to EWS-FLI1^high^ (no dox) cells.

To define gene sets involved in the anti-migratory activity of VP in EwS, we performed genome-wide transcriptome analysis by RNA-seq. We previously showed that a large number of EWS-FLI1 repressed genes are involved in migration, adhesion and the stability of the actin cytoskeleton^10^. Consistent with these results, 911 genes were found to be upregulated in A673/TR/shEF in response to EWS-FLI1 knockdown (logFC > 0.7, p < 0.05; Venn diagram: UP). Treatment with 500 nM VP significantly decreased expression of 370 genes under EWS-FLI1^low^ conditions (logFC > 0.7, p < 0.05; Venn diagram: DOWN) (Fig. 5a). Of these, 93 genes were among EWS-FLI1-suppressed genes, suggesting that VP treatment reversed their gene expression under EWS-FLI1^low^ conditions (Fig. 5b). This result was validated by qPCR of four selected genes, *PLAU*, *FBNI*, *COL5A1* and *THBS1* (Supplemental Fig. 3C). The EWS-FLI1-anticorrelated gene set affected by VP treatment was significantly enriched in the terms “migration”, “signalling to the extracellular matrix” and “cytoskeletal processes” (Fig. 5c). This finding is in line with the observed phenotypic effects on EwS plasticity upon VP treatment.

### Verteporfin reduces stress fibre and focal-adhesion formation

The transition from proliferative EWS-FLI1^high^ cells to migratory EWS-FLI1^low^ cells is characterized by profound cytoskeletal rearrangements. In order to test whether VP induces changes to cytoskeletal organization, we checked for stress fibre (F-actin) and focal-adhesion formation by confocal immunofluorescence microscopy in VP-treated EWS-FLI1^low^ cells (Fig. 5d). Consistent with earlier observations^5,7^, F-actin fibres were weak and poorly present in the EWS-FLI1^high^ state, but strongly increased upon knockdown of EWS-FLI1 by dox treatment. Upon incubation with 500 nM VP, EWS-FLI1^low^ cells exhibited thinner and much less abundant actin stress fibres similar to EWS-FLI1^high^ cells. Focal adhesions, which concentrate at cell protrusions of migratory cells^34,35^, were found to be weak and diffusely distributed in the presence of EWS-FLI1. In the migratory EWS-FLI1^low^ state, however, a strong re-localization of focal-adhesion clusters at the cell edges was observed (Fig. 5d; see also Chaturvedi et al.^7^). In contrast, VP-treated EWS-FLI1^low^ cells showed weaker and diffuse paxillin expression as compared to the DMSO control (Fig. 5d, e). At a concentration of 5 nM VP, similar effects on stress fibres and focal adhesions were visible, but less reproducible as upon treatment with 500 nM VP (Supplemental Fig. 7).

### Verteporfin reduces EwS lung metastasis in vivo

**Fig. 6:**
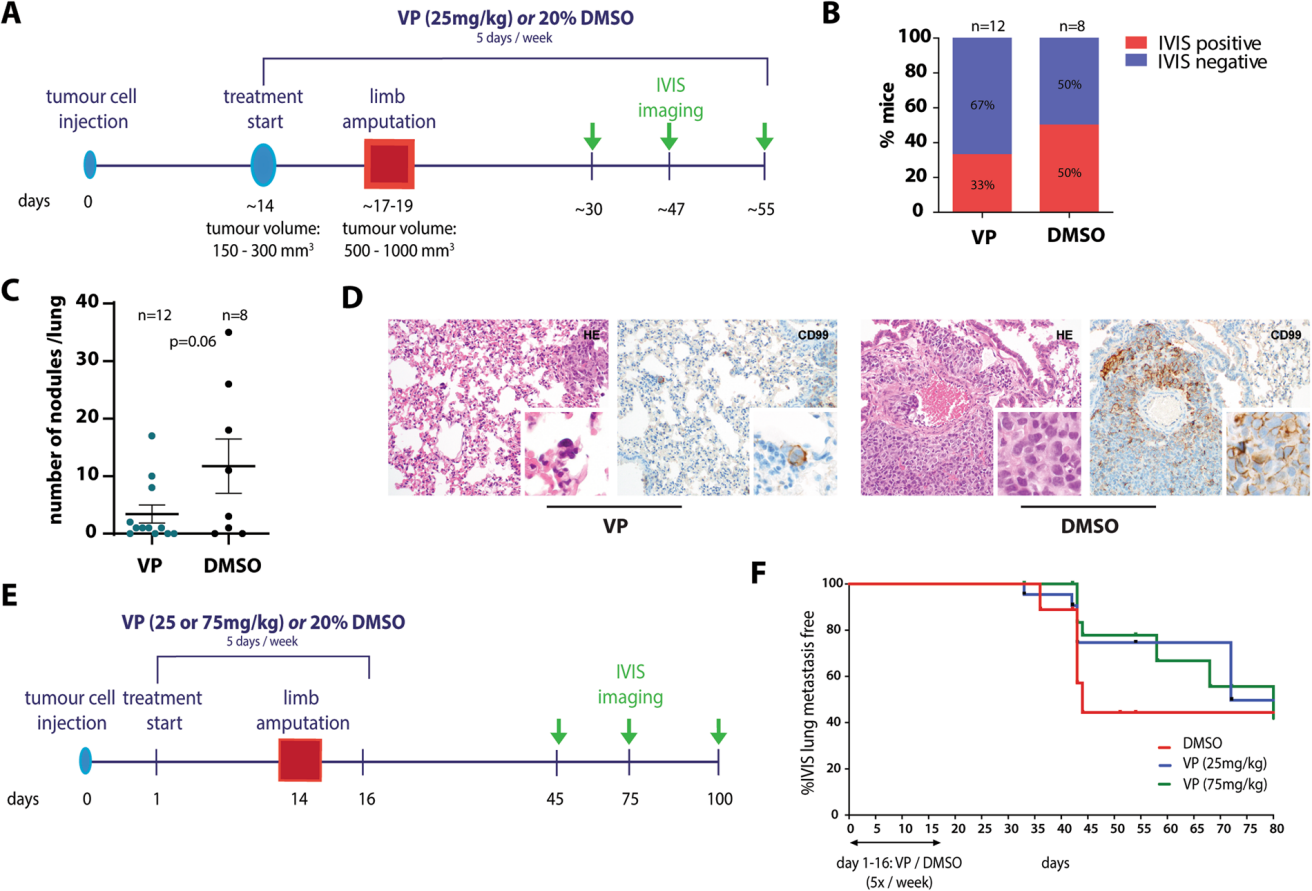
Verteporfin treatment reduces EwS lung metastasis in a mouse xenograft model. **(a)** Experimental setting 1: set-up and VP treatment scheme. Luciferase-expressing TC71 cells were injected into the tibial crest of mice. Intra-peritoneal injections of VP (25 mg/kg) or solvent control (20% DMSO) started once tumours reached a specific size. When tumours reached a volume of 150–300mm^3^, tumour-bearing limbs were amputated and VP and control treatments (5 days/week) were continued for a maximum of 35 days. **(b)** Proportions of mice with IVIS-detectable pulmonary metastases per treatment group. **(c)** Number of histopathologically detectable tumour nodules in lung sections of control- and VP-treated mice based on evaluation of H&E and CD99 stainings. The mean number ±s.e.m. of tumour nodules per condition is shown. P value was calculated by two-tailed Student’s t-test. **(d)** Exemplary H&E and CD99 stainings showing a reduced size of EwS lung metastatic nodules (200x magnification, inserts: 600x magnification). **(e)** Experimental setting 2: set-up and VP treatment scheme. As for setting 1 in (A), luciferase-expressing TC71 cells were injected, but VP (25 mg/kg or 75 mg/kg) and control treatments were started one day after tumour cell injection and stopped two days after limb amputation. **(f)** Lung metastasis free survival of control- and VP-treated mice from experimental setting 2. Although data are not statistically significant, VP-treated mice show a delay in metastatic on-set.

Our in vitro findings encouraged us to perform a pilot study testing the effect of VP treatment on metastasis in an EwS xenograft limb amputation model. Luciferase-expressing TC71 cells were injected into the tibial crest of mice and the effect of VP was tested in two different experimental set-ups. For experimental setting 1 (Fig. 6a), mice received VP (25 mg/kg/day) or 20% DMSO injections 5 times a week once palpable tumours were formed and treatment was continued until the end of the experiment. All mice underwent amputation of the tumour-bearing limb once the tumour reached a volume of ~500–1000mm^3^. VP treatment was generally well tolerated. No differences in weight gain of the animals were observed between DMSO-control and VP-treated groups and pre-surgical VP treatment did not significantly affect primary tumour growth (Supplemental Fig. 8A). While the control group (n = 8) developed local recurrences at the amputation site in 75% and lung metastases in 50% of mice, the VP-treated group (n = 12) showed recurrences at the surgical site in only 50% and lung metastases in 33% of mice (Fig. 6b). Histological evaluation of all lungs by CD99, a marker for EwS, and H&E stainings at the end of the experiment confirmed a drastic reduction in the number of pulmonary tumour nodules (mean ± SD: 11.8 ± 13.4 (DMSO) vs 3.4 ± 5.4 (VP)) (Fig. 6c, d). Tumour recurrence and metastases did neither correlate with tumour volume at the beginning of pre-surgical treatment or at amputation, nor with the number of pre-surgical drug treatments (data not shown). In experimental setting 2, VP treatment (25 or 75 mg/kg/day) was already initiated one day after tibial tumour cell implantation to target early metastatic events and was terminated two days after limb amputation (Fig. 6e). Here, a dose-dependent decrease in local recurrences at the amputation site and delayed formation of pulmonary metastases was observed in VP-treated mice (median lung metastasis free survival: 72 and 99 days for 25 and 75 mg/kg/day VP, respectively, versus 44 days for DMSO treatment) (Fig. 6f). Again, no effect of VP treatment on weight gain and primary tumour growth was observed (Supplemental Fig. 8B). Although the sample sizes were not large enough for definite conclusions and none of the results achieved statistical significance, the fact that all three experimental conditions led to either reduced or delayed metastasis formation, suggests that pre-surgical VP treatment can reduce the potential of EwS tumours to disseminate and metastasize.

Taken together, we provide evidence that pharmacologic inhibition of YAP/TAZ/TEAD with VP specifically inhibits migration and invasion of EWS-FLI1^low^ cells and reduces EwS metastatic potential.

## Discussion

A prerequisite for the initiation of metastasis is the cell’s ability to transform from a highly organized into a loose, migratory phenotype. In EwS, our previous findings indicated that the MRTFB-YAP/TEAD network hub is activated upon migratory EWS-FLI1^low^ conditions. In addition, we here provide evidence for EWS-FLI1 anticorrelated expression of TAZ in two of three EwS cell lines and generally in a cohort of EwS primary tumours.

There are several possible mechanisms for TAZ activation upon EWS-FLI1^low^ conditions: The TAZ promoter region contains binding sites for ETS, FOXO and SRF transcription factors and has been previously reported bound by ETS factors ETV1, 4, and 5 in prostate cancer^36^. Interrogating published EWS-FLI1 ChIP-seq data^37,38^, we find a FLI1 peak at the TAZ promoter of EwS cell lines, which however persisted upon EWS-FLI1 knockdown and therefore needs to be qualified as unspecific. Moreover, activation of TGFβ signalling was shown to selectively upregulate TAZ transcription through activation of the transcriptional SRF co-activator MRTF^39^, which we have previously reported to translocate to the nucleus upon EWS-FLI1 knockdown in EwS cells^10^. Further, we previously reported stabilization and nuclear translocation and activation of FOXO1 upon EWS-FLI1 knockdown leading to re-expression of a subset of EWS-FLI1 repressed genes, among them TAZ^40^. It was beyond the scope of this study to clarify which of these mechanisms, or combination thereof, accounts for the observed TAZ regulation in EwS. However, as activation of Hippo pathway downstream components has been linked to metastasis and cancer growth in several tumour types, this study aimed at exploring the potential effect of YAP/TAZ inhibition by VP on EwS mechanisms of metastasis.

VP is a FDA approved photosensitizer for treatment of neovascular macular degeneration. During photodynamic therapy, VP has to be activated by nonthermal red light for formation of cytotoxic oxygen species, which leads to local cell and tissue damage^41^. Independent of VP’s light-activated function, it is widely used experimentally to inhibit the interaction between YAP and TEAD. As this activity does not require generation of reactive oxygen radicals, we protected VP and control treated cells from ambient light in all our experiments. Under these conditions, low nanomolar concentrations of VP strongly prohibited cell migration and invasion of EWS-FLI1^low^ cells without affecting proliferation and cell viability in vitro and tumour growth in vivo. VP also strongly inhibited migratory and invasive capacities of TC32/223 cells, where we did not find TAZ to be transcriptionally upregulated upon EWS-FLI1^low^ conditions, suggesting that the inhibitory activity of VP does not solely depend on interfering with increased expression levels of TAZ. Mechanistically, our PLA and co-IP data provide evidence that VP specifically blocks binding of both YAP and TAZ to TEAD resulting in downregulation of a metastasis-promoting subset of EWS-FLI1 anticorrelated genes, among them a substantial number of collagen genes. Collagen is a major constituent of the extracellular matrix (ECM), which is not solely important as a cell scaffold but also for the transmission of signals from the extracellular environment to cells^42,43^. Concomitantly, VP treatment reduced stress fibre and focal-adhesion formation and therefore morphologically reversed invasive EWS-FLI1^low^ cells into a less migratory EWS-FLI1^high^-like state. Stress fibres connect to the ECM and communicate with neighbouring cells via integrin receptor enriched focal adhesions, and provide force for cellular contractility in processes such as migration and invasion^44^. Thus, by regulating expression of collagen genes and stress fibre formation, EWS-FLI1 modulates communication between tumour cells and the ECM, invasion and migration.

However, the utility of VP as a specific YAP/TAZ/TEAD complex inhibitor is still debated. Although VP modifies the transcriptional output of the YAP/TAZ pathway in several cancer types, the physical association of VP with TEAD or YAP has not been proven yet. Among non-photo-induced biological activities, VP also exerts YAP/TAZ-independent proteotoxic activity by inducing the formation of cytotoxic high molecular weight complexes including the autophagosome component p62 (SQSTM1)^45^ and the transcription factor STAT3^46^. Furthermore, VP was reported to act as an autophagy inhibitor^47^ and to promote lysosomal compartment dysregulation^48^. Thus, although we could clearly demonstrate that VP inhibits YAP/TAZ/TEAD complex formation and transcriptional activity in EwS, we cannot exclude that the observed anti-metastatic effect of the drug involves additional mechanisms. Nevertheless, its anti-migratory activity on EwS cells at low concentrations may make it a promising candidate for combination treatment with cytotoxic standard-of-care drugs to potentially prohibit tumour dissemination, especially in patients diagnosed with localized disease. It should be mentioned, that VP has not yet been used as continuous systemic treatment in patients and outside of ophthalmology, and even though no apparent toxic side effects were observed in our mouse studies, safety and health issues such as increased skin and eye photosensitivity may limit its applicability. Our study should therefore be regarded primarily as a proof of concept for YAP/TAZ/TEAD pathway inhibition in EwS metastasis rather than a preclinical drug testing effort.

Several compounds have been described to interfere with YAP/TAZ signalling at various levels (reviewed in^49^), but to date no direct inhibitors other than VP have been described. However, several YAP/TAZ/TEAD inhibitory small molecules are currently under development^50^ and may hold promise for therapeutic application to EwS patients in the future.

Moreover, fluctuations in EWS-FLI1 levels are not the only mechanism known to initiate EwS dissemination^51^, and several candidates such as chemokine receptor CXCR4 signalling^52^, chromatin modifiers^53^, cadherins^54^, micro-RNAs^55^ and interactions with the tumour microenvironment including glucocorticoids^56,57^ have been proposed. In our in vivo proof-of-principle experiments, reduction in onset and number of lung metastases depending on dose and timing of VP treatment did not achieve statistical significance. However, considering metastasis as a complex multi-step process, it is not surprising that inhibition of YAP/TAZ/TEAD complex formation by VP alone did not completely block metastasis, but rather resulted in a delay of the metastatic onset. Pharmacokinetic limitations such as VP’s short half-life and rapid elimination in the bile^58^ as well as additional metastasis-promoting mechanisms may have been responsible for not completely prohibiting metastasis in our tested experimental settings. In addition, as stochastic fluctuations in EWS-FLI1 have been proposed to lead to oscillations between migratory and proliferative phenotypes of EwS cells, it is possible that not all tumour cells were hit during their migratory state by the VP treatment pulses. However, as all three investigated VP treatment conditions revealed a trend towards metastasis reduction, further schedule and dose optimization is justified to validate our preliminary in vivo results.

In conclusion, this study suggests that YAP/TAZ inhibitors may be considered for combination therapy with cytotoxic standard-of-care chemotherapy to prevent onset of the metastatic cascade.

## Materials and Methods

### Cell culture

A673/TR/shEF and shSKE-17T cell clones harbouring dox-inducible sh-EWS-FLI1 constructs were derived from A673 and SK-N-MC parental cell lines, and were kindly provided by J. Alonso (Instituto de Investigación de Enfermedades Raras, Madrid, Spain) and O. Delattre (Institute Curie, Paris, France)^5,59^. SKshFli1#3 cell clone carrying a dox-inducible shRNA construct targeting the 3’UTR of FLI1 (5’-TTATTCATCTCTTTGTTCAGGT-3’; pRSIT-U6Tet-shRNA-PGK-TetRep-2A-GFP-2A-puro vector plasmid; Cellecta Inc., Mountain View, CA, USA) was generated from SK-N-MC parental cell line. TC32/223 cells were a kind gift of David McFadden from UT Southwestern, Houston, Texas. Knockdown of EWS-FLI1 by shRNA induction was achieved by addition of 1 μg/ml dox (A673/TR/shEF, shSKE-17T, TC32/223) or 100 ng/ml dox (SKshFli1#3) to the medium. Cell lines were authenticated by STR profiling and were regularly screened for mycoplasma (Mykoalert detection kit; Lonza, Basel, Switzerland).

### Boyden Chamber Migration Assay

For EWS-FLI1 knockdown, cells were pre-treated with 1 µg/ml dox for 24 h (A673/TR/shEF, TC32/223) or 7 days (shSKE-17T). For transient EWS-FLI1 knockdown in TC71 cells, 4 µg shEF1∆RV30^60^ were transfected, followed by a 72 h puromycine (1 µg/mL) selection. Cells were then treated with VP (SML0534; Sigma-Aldrich/Merck, Darmstadt, Germany) or DMSO (solvent control) in the presence or absence of dox for additional 24 h. Cells were starved for 6 h before seeding 3×10^5^ (A673/TR/shEF) or 5×10^5^ (TC32/223, shSKE-17T) cells into pre-equilibrated 24-well transwell inserts (8 µm pore size, COS3422, Corning, NY, USA) and were incubated for 18 h (A673/TR/shEF), 24 h (TC32/223), 48 h (shSKE-17T) or 72 h (TC71). Subsequently, non-migrated cells were removed from the upper chamber using a cotton swab, migrated cells were fixed with 4% formaldehyde and stained with 1% crystal-violet/20% methanol. Quantification was achieved by counting of cells in five random fields (10x magnification) per replicate using ImageJ. All experiments were performed in three technical and three biological replicates.

### Proximity Ligation Assay

A673/TR/shEF cells were cultured on fibronectin-coated chamber cells and treated as described for Boyden chamber assays. Cells were then subjected to proximity ligation assays using the Duolink™ In Situ Orange Starter Kit Mouse/Rabbit (DUO92102; Sigma-Aldrich/Merck), following the manufacturer’s instructions. Fluorescent signals were detected by confocal microscopy. Used antibodies and dilutions are listed in Supplemental Table 1.

### Immunoblotting

Protein extraction and immunoblotting with antibodies listed in Supplemental Table 1 were performed according to standard procedures. Nuclear and cytoplasmic protein fractions were separated using the Nuclei EZ Prep Nuclei Isolation Kit (NUC-101, Sigma-Aldrich/Merck).

### siRNA knockdown

For silencing of YAP1 and TAZ, cells were transfected with ON-TARGETplus SMARTpool human siRNAs (Dharmacon/Horizon, Cambridge, UK) against YAP (L-012200–00–0005) or TAZ (L-016083–00–0005) using Oligofectamine reagent (58303, Invitrogen/Thermo Fisher Scientific). As a control, ON-TARGETplus non-targeting pool (D-001810–10–20) was used. After 24 h, the transfection procedure was repeated and cells were used for migration assays after additional 24 h.

### Mouse experiments

All animal studies were conducted under a protocol approved by the Georgetown University´s Institutional Animal Care and Use Committee (IACUC) in accordance with NIH guidelines for the ethical treatment of animals (approval number 2016–1174). Luciferase-expressing TC71 (1×10^6^) cells were injected into the tibial crest of 5 week old female SCID beige mice. Palpable tumours formed within 14 days. Animals were randomized into vehicle or treatment groups based on the order of retrieval from cages. Cages were housed in random order on shelves. For experimental setting 1, animals received intra-peritoneal injections of solvent (20% DMSO) or VP (D480571; eNovation Chemicals, Green Brook, NJ, USA) at a dose of 25 mg/kg per day (5 days per week) once tumours reached ~150–300mm^3^. Pre-surgical treatment continued for 3 to 7 days until tumours reached a volume of 500–1000mm^3^, at which time the tumour-bearing limb was amputated. After a recovery period of 3 days, treatments were resumed for a maximum duration of 35 days. For experimental setting 2, DMSO or VP (S1786; Selleck Chemicals, Houston, TX, USA) at a dose of 25 mg/kg or 75 mg/kg was injected one day after tumour cell injection and continued daily (5 days per week) until two days after limb amputation. Animals were monitored for tumour recurrence at the amputation site and for pulmonary metastasis by IVIS imaging. Tumour nodules in lung sections were analysed by evaluation of H&E and CD99 stainings. Pathologists analysing the tissues were not involved in the animal experiments and therefore blind to the treatment.

### Confocal microscopy

Confocal images were taken using the Leica TCS SP8 confocal microscope (Leica Microsystems, Wetzlar, Germany). For imaging of focal adhesions, cells were stained with paxillin antibody (Supplemental Table 1) and quantification was performed using ImageJ (see Supplemental Methods). F-actin fibres were stained with TRITC-phalloidin in Vectashield mounting medium (H-1600; Vectorlabs, Burlingame, CA, USA). KI67 staining (Supplemental Table 1) was performed under Boyden chamber assay conditions. KI67 positive and negative fractions among a total of 200 counted cells per condition and replicate (n = 3) was determined and normalized to the total number of counted cells.

### Statistical analysis

Statistics were calculated from three independent experiments using the Graph Pad Prism 8 software and data are represented as the mean ± s.e.m. (standard error of the mean). P-values were analysed by either using two-tailed Student’s t-test or one-sample t-test when the hypothetical mean value was set to 1. ****p ≤ 0.0001; ***p ≤ 0.001; **p ≤ 0.01; *p ≤ 0.05.

## Supplementary information

Supplemental information

Supplemental Table 1
