# Supplementary material for: YAP/TAZ inhibition reduces metastatic potential of Ewing sarcoma cells
Source: Oncogenesis. 2021 Jan 8;10(1):2. doi: 10.1038/s41389-020-00294-8 (PMC7794350; doi:10.1038/s41389-020-00294-8)
Supplement: Supplementary file 1 — Supplemental information [file 41389_2020_294_MOESM1_ESM.docx]

**Supplemental Information**


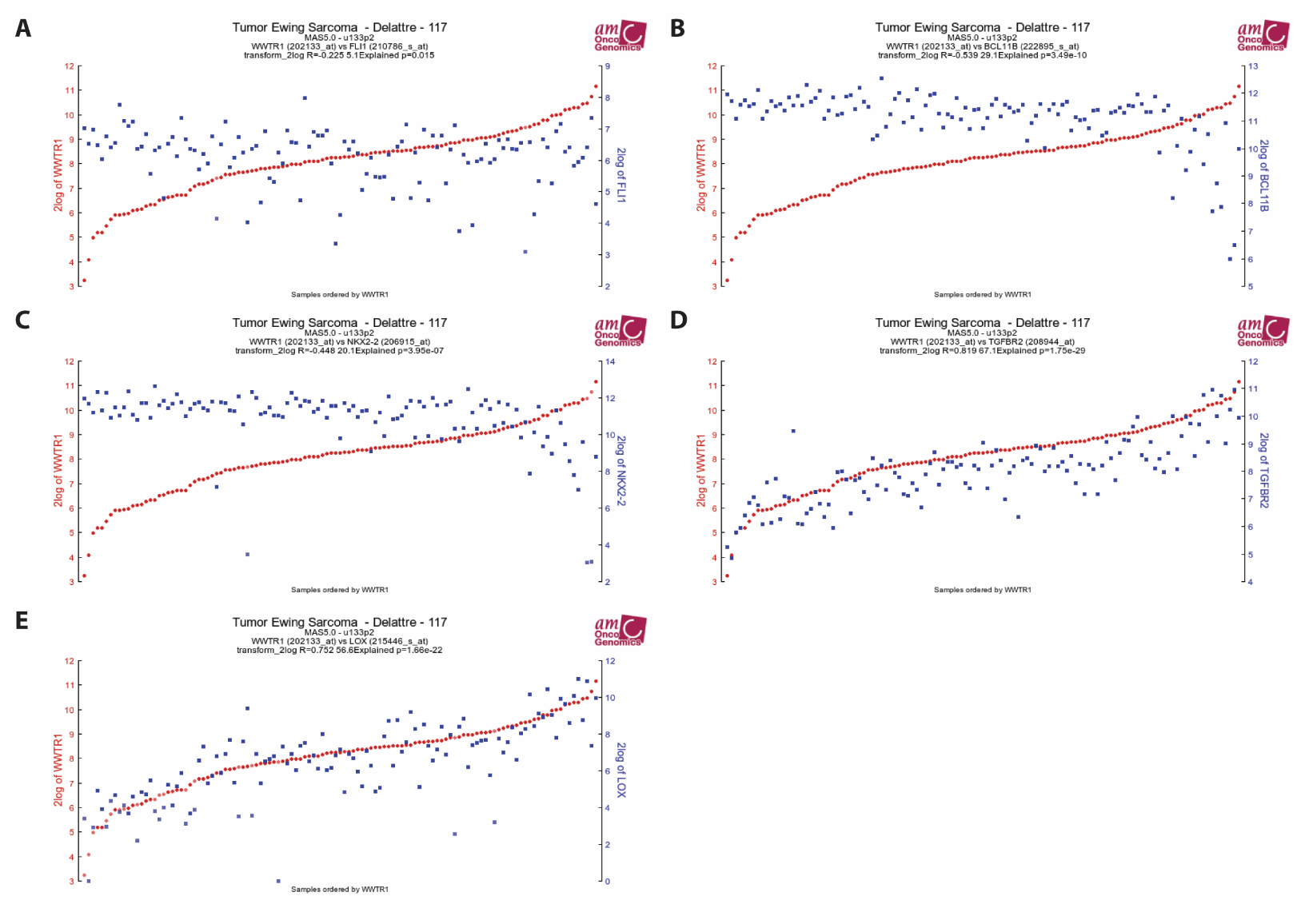
**Supplemental Figures**

**Supplemental Figure 1. TAZ expression correlates with EWS-FLI1 and its downstream targets in primary EwS tumours.**

Correlation analysis of TAZ (WWTR1) expression with **(A)** FLI1 (r=-0.225, p=0.015), **(B)** BCL11B (r=0.539, p=3.49e-10), **(C)** NKX2-2 (r=-0.448, p=3.95e-07) **(D)** TGFBR2 (r=0.819, p=1.75e-29) and **(E)** LOX (r=0.752, p=0.166e-22) in 117 primary EwS tumours (GEO ID: gse34620(1)). Data was visualized using the R2 Genomics Analysis and Visualization Platform (http://r2.amc.nl).

**
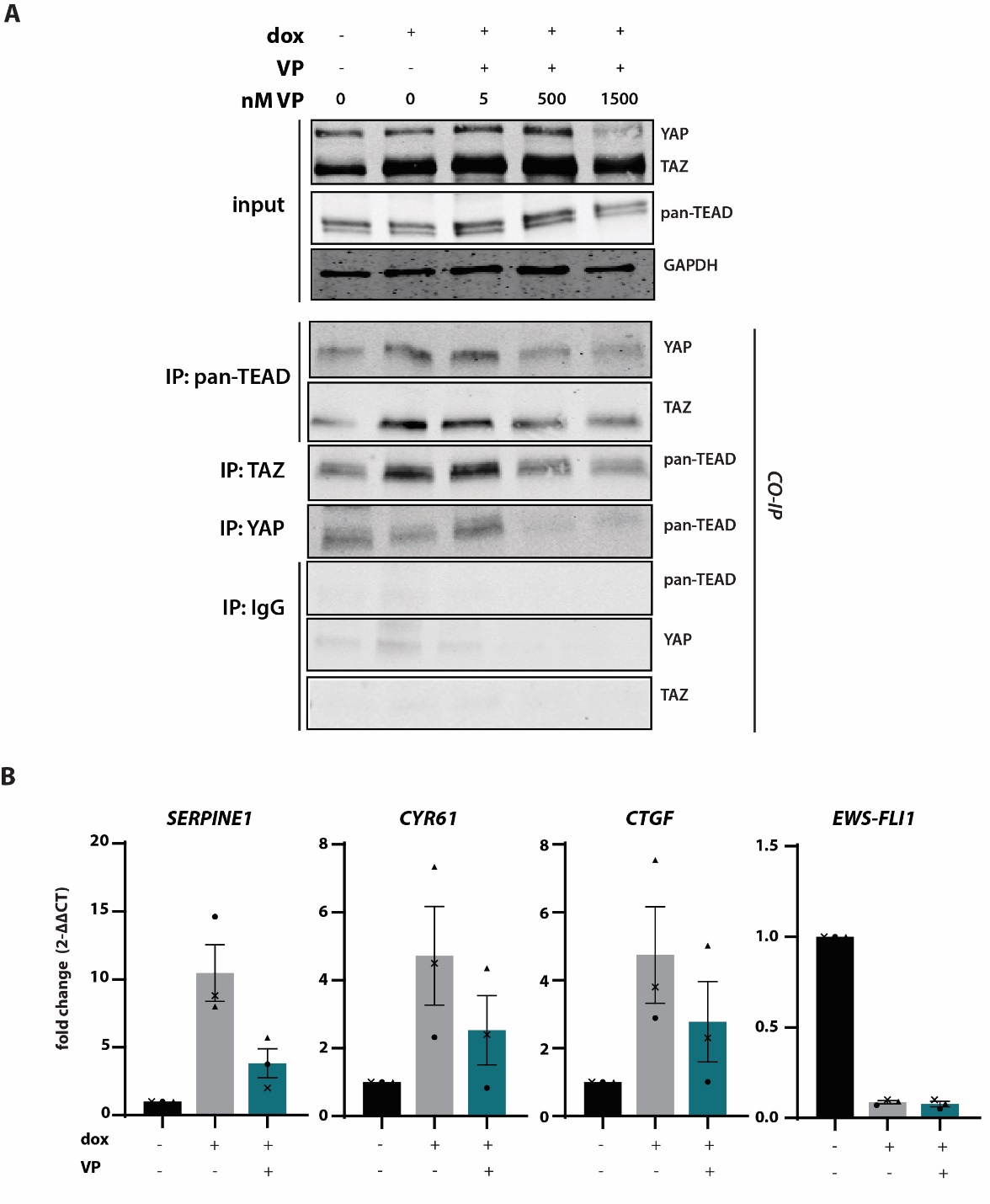
**

**Supplemental Figure 2. Verteporfin interferes with YAP/TAZ/TEAD complex formation and inhibits YAP/TAZ/TEAD target gene expression in EWS-FLI1^low^ conditions.**

**(A)** Immunoblot showing input and co-IP results of YAP, TAZ and TEAD upon EWS-FLI1^high^ (no dox) and EWS-FLI1^low^ (+dox) conditions and upon VP (5nM, 500nM, 1.5µM) treatment. One representative experiment from two biological replicates is shown. **(B)** qPCR analysis of YAP/TAZ target gene (*SERPINE*1, *CYR61*, *CTGF*) and *EWS-FLI1* expression upon EWS-FLI1^high^ (no dox), EWS-FLI1^low^ (+dox) conditions and concomitant VP or control (DMSO) treatment in A673/TR/shEF cells. Cells were pre-treated with dox (1µg/ml) for 24h and subsequently VP (500nM) was added for another 24h. Data from three independent experiments (**○, ∆, x**) is shown as expression fold change relative to no dox / +DMSO conditions. Mean fold change ± s.e.m is indicated.


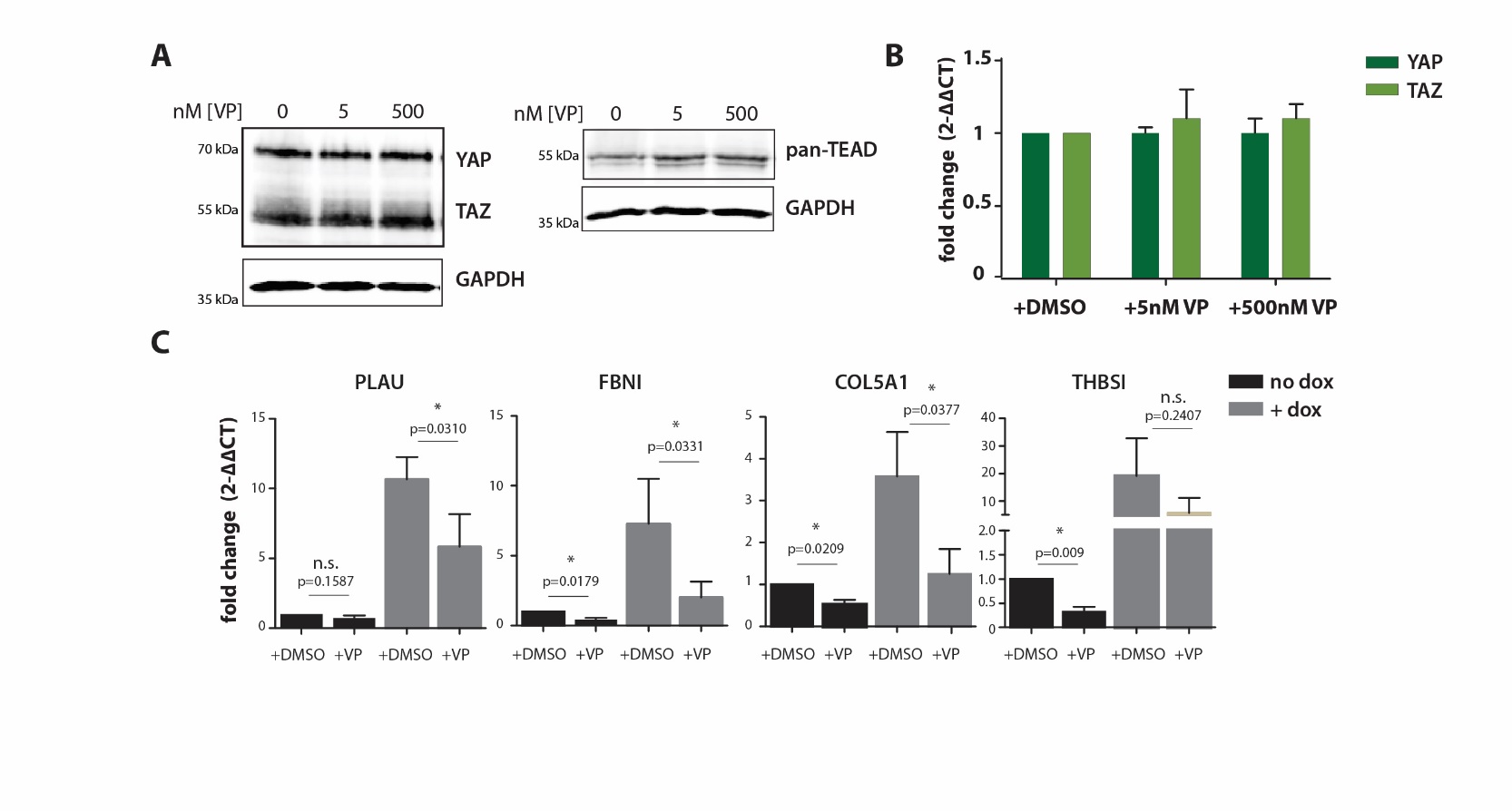
**Supplemental Figure 3. Verteporfin acts independently of EWS-FLI1 expression levels in A673/TR/shEF cells.**

**(A)** Representative Western blot showing YAP, TAZ and pan-TEAD expression levels upon 24 hours of VP treatment of EWS-FLI1^high^ cells. Similar to EWS-FLI1^low^ conditions (see Figure 2), protein expression of YAP/TAZ/TEAD complex components are not affected by VP treatment. **(B)** qPCR analysis of YAP and TAZ mRNA levels upon same treatments as in (A). **(C)** VP affects expression of selected EWS-FLI1 suppressed genes. Relative mRNA abundance of selected cytoskeletal genes (*PLAU*, *COL5A1*, *FBN1*, *THBS1*) under EWS-FLI1^high^ (no dox) and EWS-FLI1^low^ (+dox) conditions upon 500nM VP treatment analyzed by qPCR. As A673/TR/shEF cells migrate also in the EWS-FLI1^high^ state (see Figure 3), the inhibitory effect of VP treatment was also observed under control conditions. qPCR data in (B) and (C) are expressed as mean fold change ± SD relative to no dox/DMSO control of three independent experiments. Statistics for (C) were calculated by One sample t-test, specifying the hypothetical mean value as 1 (no dox/DMSO vs no dox/500nM VP), or two-tailed unpaired t-test (+dox/DMSO vs +dox/500nM VP). *p≤0.05.


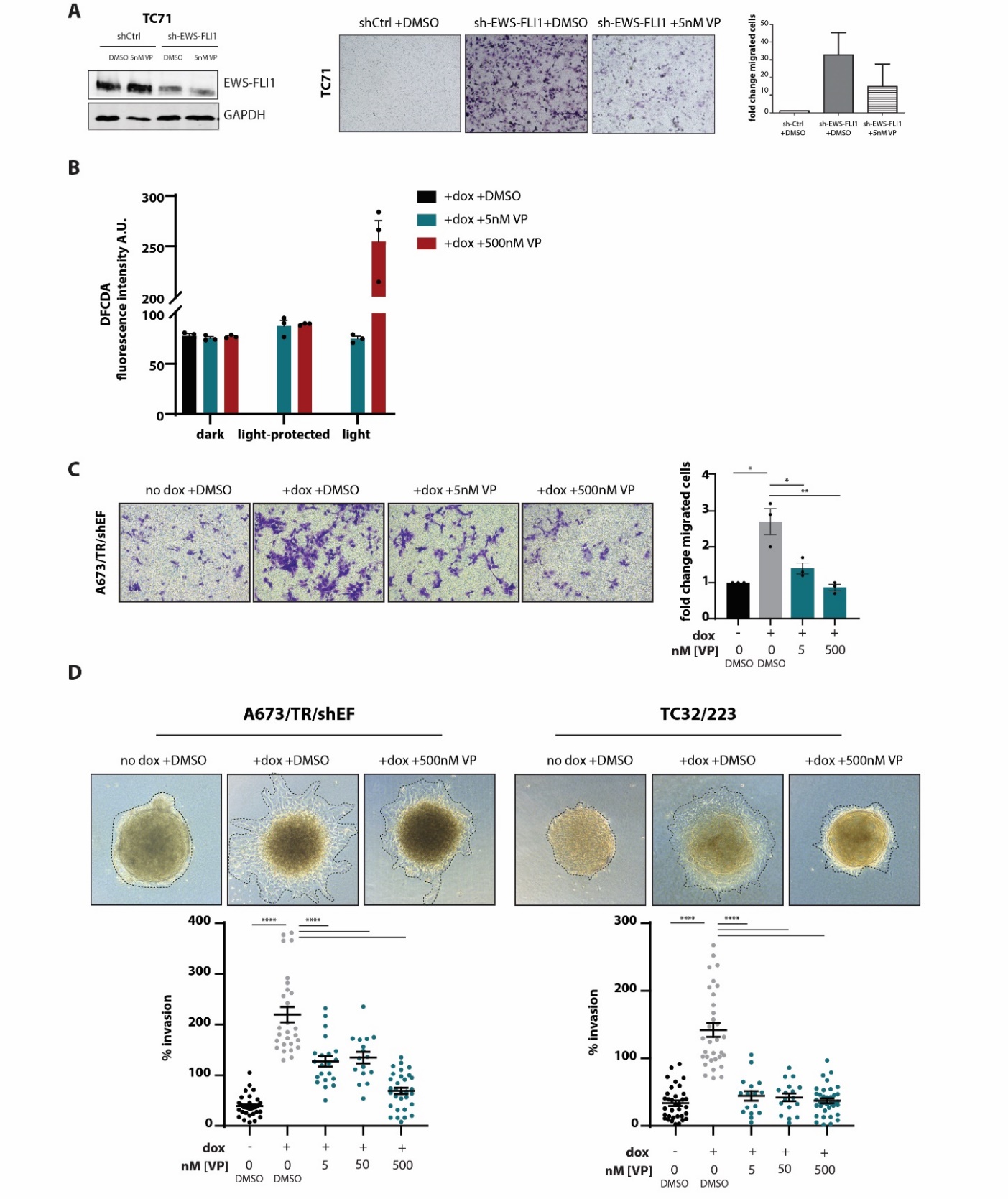
**
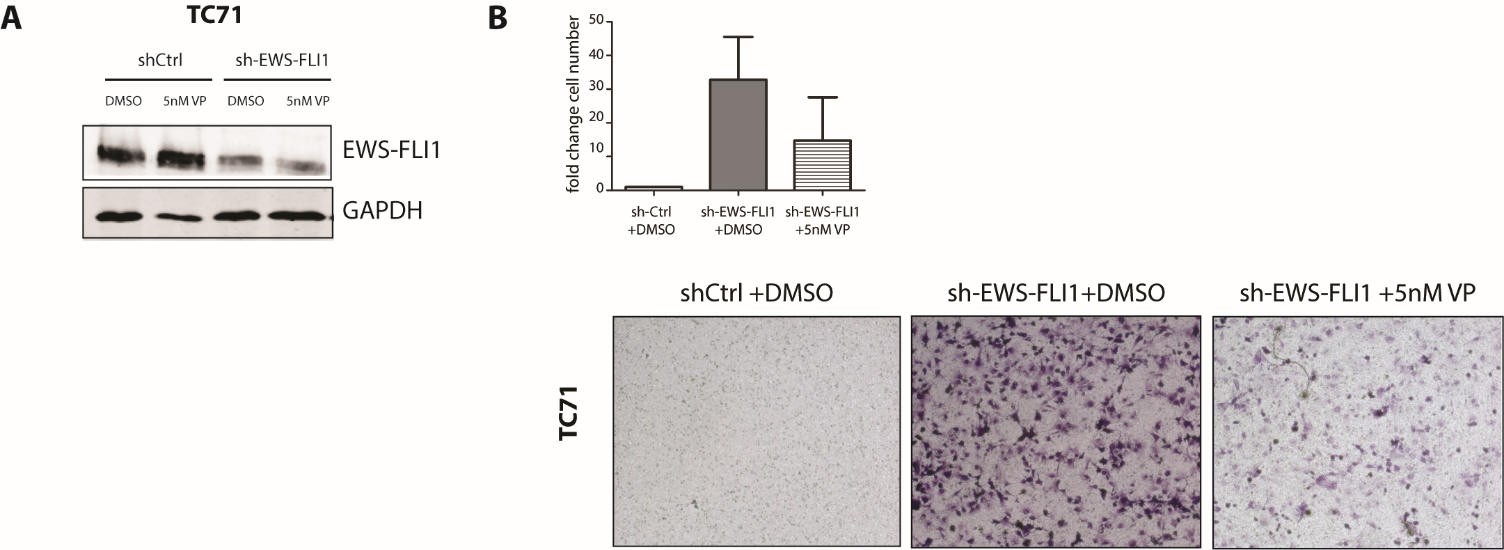
Supplemental Figure 4. Verteporfin prohibits EwS cell migration and invasion under EWS-FLI1^low^ conditions with only minor contribution of light-induced ROS.**

**(A)** Boyden chamber migration assay of stably transfected TC71 cells treated with 5nM VP or DMSO (solvent control) for 24h. Comparable to dox-inducible EWS-FLI1 knockdown cell lines (see Figure 3), TC71 cells transfected with sh-EWS-FLI1 migrated stronger as compared to control (shCtrl), which was inhibited by 5nM VP treatment. Quantification was achieved by manual counting of cells in five random fields (10x magnification) per replicate. Counted cell numbers were normalized to shCtrl- transfected cells treated with DMSO. Experiments were performed in three technical and two biological replicates. A representative immunoblot showing efficiency of transient EWS-FLI1 knockdown in TC71 EwS cells is shown. **(B)** General ROS production under different light conditions was assessed by CM-H2DCFDA fluorescence using flow cytometry. Experiments were either performed in darkness (“dark”), in an environment protected from artificial light illumination (“light protected”) or under unprotected ambient light conditions (“light”). Mean intensities ± s.e.m. of 3 independent experiments is shown. **(C)** Boyden chamber migration assay of A673/TR/shEF cells (±dox) treated with VP in complete darkness. Experimental settings and data analysis are comparable to migration assays shown in main Figure 3. Experiment was performed in three technical and three biological replicates. **(D)** Collagen I multicellular spheroid invasion assay of A673/TR/shEF and TC32/223 spheroids under EWS-FLI1^high^ (no dox) and EWS-FLI1^low^ (+dox) conditions, generated by the hanging drop method (72h) in presence or absence of different VP concentrations. Exemplary spheres from three independent experiments are shown. Dashed lines mark the invasive front 24 hours after sphere embedding. Invaded areas were measured by ImageJ and put in correlation to initial sphere size (% invasion of initial sphere size). Statistics were calculated by two-sided, unpaired Student’s t-test. ****p≤0.0001.

**
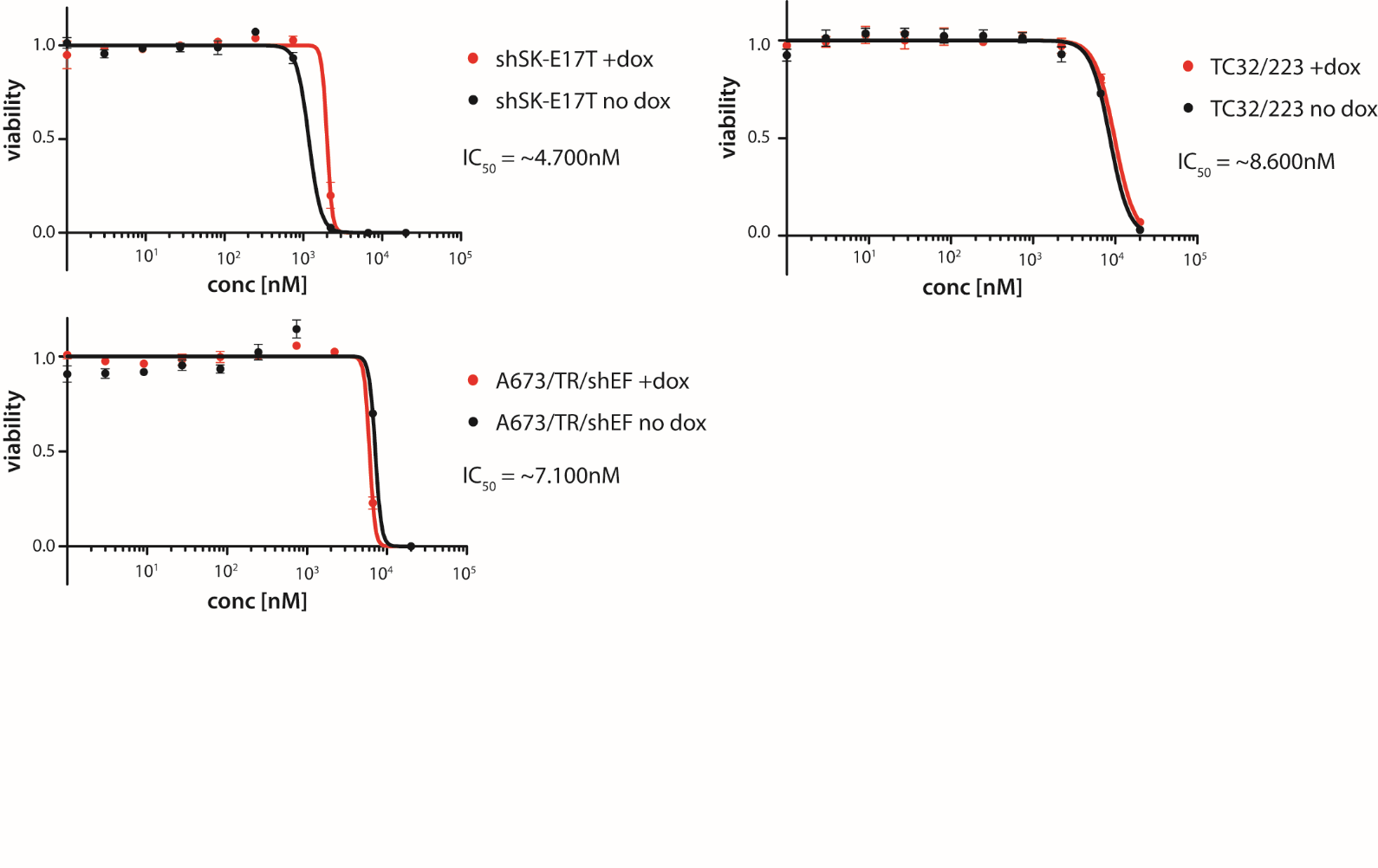
**

**Supplemental Figure 5. Verteporfin kills EwS cells at concentrations in the micromolar range, significantly higher than required to block their migration.**

A673/TR/shEF, shSK-E17T and TC32/223 EwS cell lines were treated with serial dilutions of VP for 72h under EWS-FLI1^high^ (no dox) and EWS-FLI1^low^ (+dox) conditions. All experiments were performed in the dark. No major differences in drug toxicity between EWS-FLI1^high^ and EWS-FLI1^low^ states were observed. IC_50_ values of “no dox” conditions are indicated.


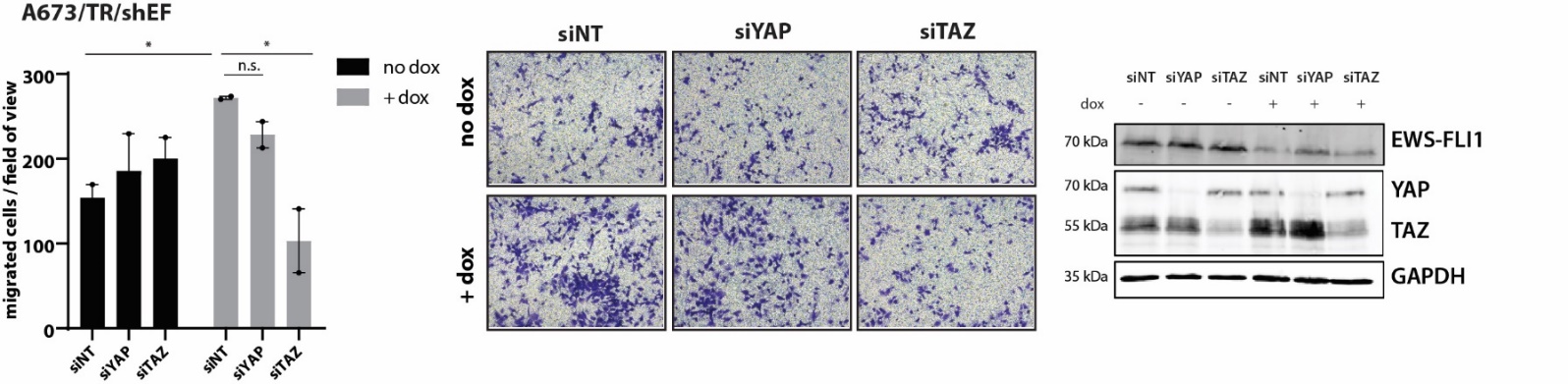
**Supplemental Figure 6. Effect of YAP and TAZ knockdown on the migratory capacity of EWS-FLI1^low^** **A673/TR/shEF** **cells.**

Transwell assays upon individual silencing of YAP (siYAP) or TAZ (siTAZ), compared to siNT-transfected cells, under EWS-FLI1^high^ and dox-induced EWS-FLI1^low^ conditions. Average number of migrated cells ± s.e.m. of two biological replicates, each performed in technical triplicates, is presented. A representative Western blot is shown to verify the efficient silencing of YAP and TAZ and dox-induced EWS-FLI1 knockdown.

**
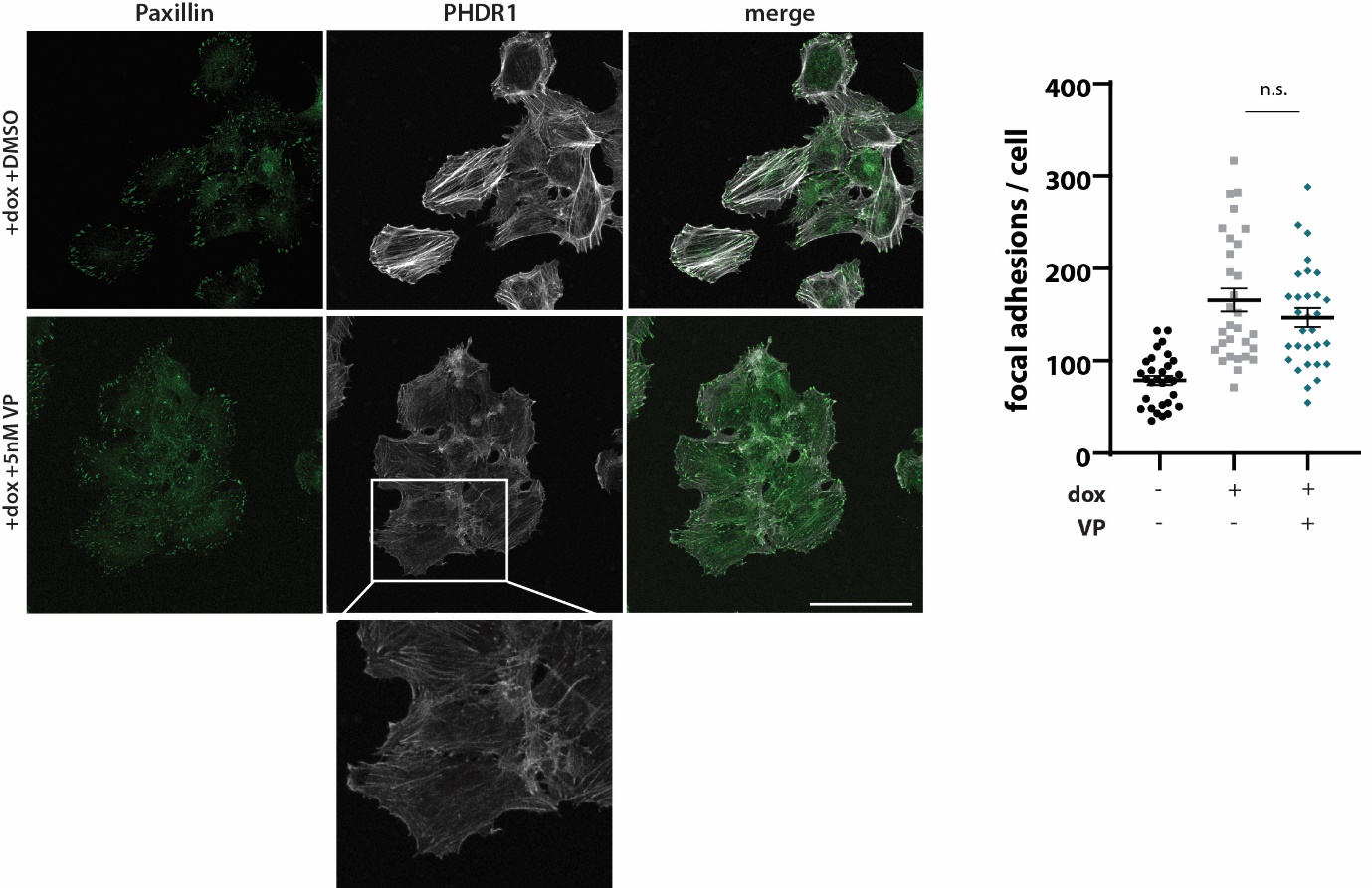
**

**Supplemental Figure 7. Verteporfin treatment affects focal adhesions and actin fibers.**

Confocal immunofluorescence imaging of focal adhesions (stained with paxillin antibodies) and F-actin fibers (stained with TRITC-phalloidin). A673/TR/shEF cells were treated for 24h with 1µg/ml dox to induce EWS-FLI1 conditions, and then incubated with 5nM VP or DMSO for another 24h. Representative images and quantification of two biological replicates are shown (scale bar: 100µm, selection: 146% magnification). Quantification of paxillin-positive foci per cell, normalized to respective cell size, was performed as described in Supplemental Methods. In contrast to 500nM VP treatment (see Figure 5), 5nM VP treatment was less effective in downregulation of focal adhesions.

**
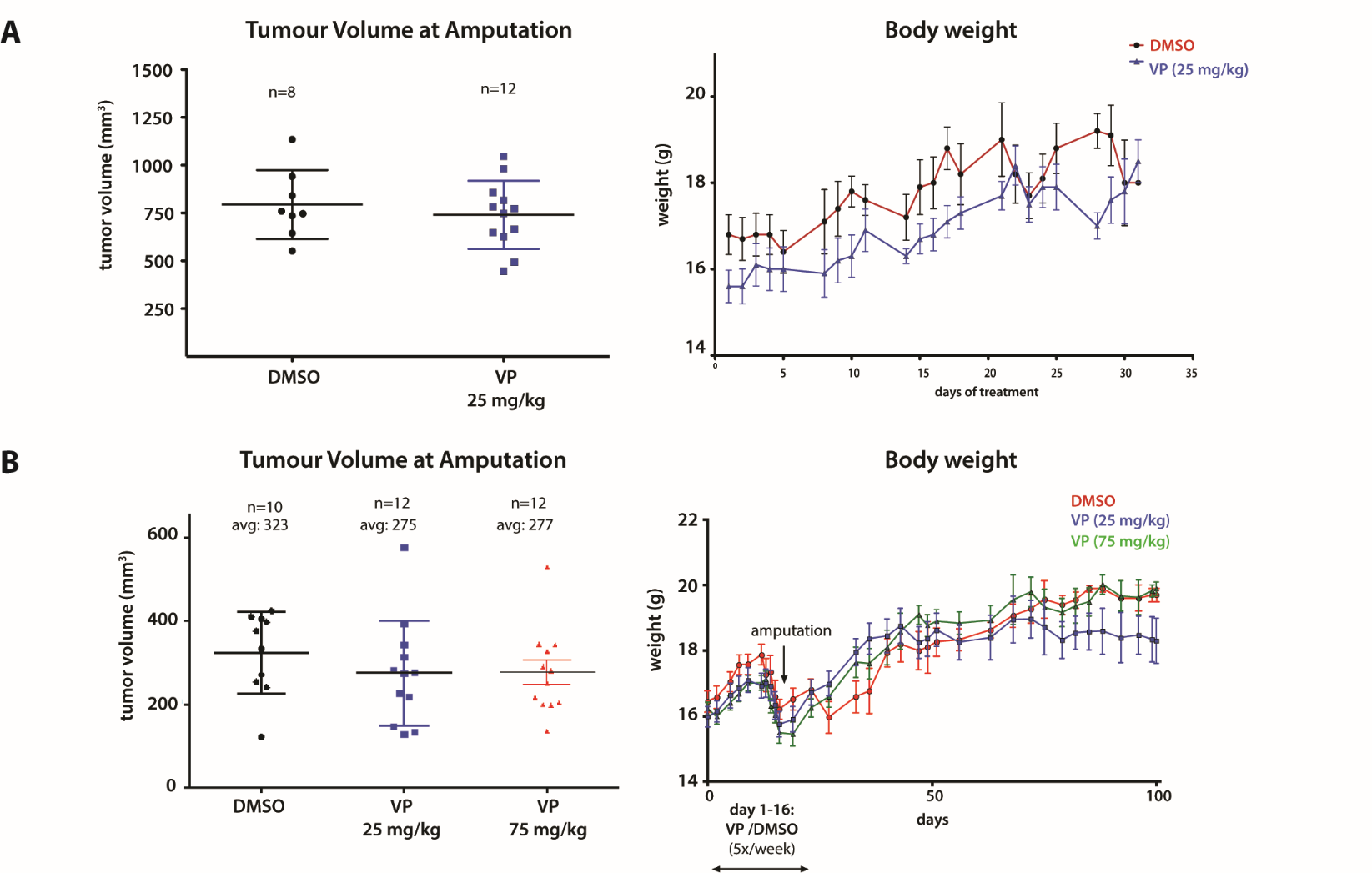
**

**Supplemental Figure 8. Verteporfin treatment does not affect body weight gain or primary tumour growth *in vivo*. (A)** Primary tumour volume (left) and body weight gain of VP- and control-treated mice during experimental setting 1 (see Figure 6A). Tumour volume was measured right before amputation; body weight gain was traced on all treatment days. **(B)** Same measurements as in (A), but for experimental setting 2 (see Figure 6E-F).

**Supplemental Methods**

Tumour Spheroid Collagen Invasion Assay

Generation of multicellular spheroids and invasion assays were performed as described previously (2). Briefly, A673/shEF/TR and TC32/223 spheroids were generated by the manual hanging drop technique. 6000 cells were seeded in 20 µl drops of standard cell culture medium (± 1µg/ml dox, ± VP) and 72h after natural aggregation by gravity, spheroids were transferred to 24 well plates and embedded in 60µl collagen mixture (2.2 mg/ml rat collagen, 1179179001, Merck). Spheres were monitored after 24h and invaded areas relative to initial sphere size were calculated using ImageJ.

Cellular ROS measurements

For cellular ROS measurements, the general oxidative stress indicator CM-H2DCFDA (C6827, Thermo Fisher Scientific, Waltham, MA, USA) was used. A673/TR/shEF cells were pre-treated with 1µg/ml dox for 24 hours and then VP was added for another 24 hours. For different light conditions, the experiments were performed either in a dark room (“dark”) or in a standard cell culture room with all artificial lights turned off (“light-protected”) or turned on (“light”). 24 hours after start of VP treatment, cells were stained with 1µM H2DCFDA for 30 minutes at 37°C and analyzed by flow cytometry.

Co-Immunoprecipitation

A673/TR/shEF cells were treated with 1µg/mL dox for 24h and then treated with different concentrations of VP (-/+dox) for another 24h before harvesting. Antibodies (**Supplemental Table 1**) were crosslinked to Protein G Dynabeads (50µl/IP) (Thermo Fisher Scientific) over night, and subsequently incubated with protein lysates from approximately 10^7^ cells per IP. Immunoblots of input protein extracts and immunoprecipitates were probed with the indicated antibodies.

Cell Viability Assay

Cells (1000 - 2000 cells/well) were seeded in 96-well plates in triplicates and left to adhere overnight. VP (0.003µM to 20µM serial dilutions) was applied for 72h. In case of concomitant EWS-FLI1 knockdown, the induction with dox was started 24h prior to VP treatment and cells were kept in dox until the readout. Cell viability was quantified using the CellTiter-Glo® Cell Viability Assay (Promega). IC50 values were determined by fitting a dose-response curve to the data points using nonlinear regression analysis utilizing the GraphPad Prism 8 software (Graph Pad Prism Software Inc., La Jolla, CA, USA).

qPCR

Total RNA was isolated with the Qiagen RNeasy kit (Qiagen, Hilden, Germany) and cDNA was generated using M-MLV Reverse Transcriptase (Promega, Madison, WI, USA). qPCR was performed using Maxima-SYBR green/ROX master mix (Thermo Fisher Scientific). CT values were normalized to GAPDH. Relative expression values were analysed by the 2^-∆∆CT^ method (3) and are indicated as fold change relative to control conditions. All primer sequences are provided in Supplemental Table 1.

Measurement and quantification of focal adhesions

To achieve quantification of paxillin - positive focal adhesions, we followed a previously published protocol with slight modifications (4). In brief, 30 randomly selected cells per experimental condition were imaged (40x magnification) using a standard fluorescence microscope. For automated image processing, pictures were processed using the following macro installed in ImageJ:

run("8-bit");

run("Subtract Background...", "rolling=50 sliding");

run("CLAHE ", "blocksize=19 histogram=256 maximum=6");

setAutoThreshold("Default dark");

//run("Threshold...");

setThreshold(39, 255);

//setThreshold(39, 255);

setOption("BlackBackground", true);

run("Convert to Mask");

Next, cell areas were manually selected and particles were counted using the “analyze particles” command, according to pre - defined parameters for focal adhesions (size = 10 - infinity; circularity = 0.00 - 0.99). Number of focal adhesions were normalized to the respective cell area.

**Supplemental References**

1. Postel-Vinay S, Veron AS, Tirode F, Pierron G, Reynaud S, Kovar H, et al. Common variants near TARDBP and EGR2 are associated with susceptibility to Ewing sarcoma. Nature genetics. 2012;44(3):323-7.

2. Cisneros Castillo LR, Oancea AD, Stüllein C, Régnier-Vigouroux A. Evaluation of Consistency in Spheroid Invasion Assays. Scientific reports. 2016;6:28375.

3. Schmittgen TD, Livak KJ. Analyzing real-time PCR data by the comparative C(T) method. Nat Protoc. 2008;3(6):1101-8.

4. Horzum U, Ozdil B, Pesen-Okvur D. Step-by-step quantitative analysis of focal adhesions. MethodsX. 2014;1:56-9.
